# Tibial dyschondroplasia is highly associated with suppression of tibial angiogenesis through regulating the HIF-1α/VEGF/VEGFR signaling pathway in chickens

**DOI:** 10.1038/s41598-017-09664-6

**Published:** 2017-08-22

**Authors:** Shu-cheng Huang, Mujeeb Ur Rehman, Yan-fang Lan, Gang Qiu, Hui Zhang, Muhammad Kashif Iqbal, Hou-qiang Luo, Khalid Mehmood, Li-hong Zhang, Jia-kui Li

**Affiliations:** 1 College of Veterinary Medicine, Huazhong Agricultural University, Wuhan, 430070 People’s Republic of China; 2 Laboratory of Detection and Monitoring of Highland Animal Disease, Tibet Agriculture and Animal Husbandry College, Linzhi, 860000 Tibet People’s Republic of China; 3 Animal Science Department, Wenzhou Vocational College of Science and Technology, Wenzhou, 325006 People’s Republic of China; 4 University College of Veterinary & Animal Sciences, The Islamia University of Bahawalpur, Bahawalpur, 631000 Pakistan

## Abstract

Tibial dyschondroplasia (TD) is an intractable poultry problem that is characterized by the appearance of non-vascularized and non-mineralized cartilage masses in tibial growth plates (TGPs). However, the role of angiogenesis inhibition in the occurrence of TD remains unknown. In this study, we found that, compared to low-altitude Arbor Acres chickens (AACs), high-altitude Tibetan chickens showed higher tibial vascular distributions that were accompanied by up-regulation of hypoxia-inducible factor-1α (HIF-1α), vascular endothelial growth factor A (VEGFA) and VEGF receptors. These observations provide insights into hypoxia-induced angiogenesis, which may be related to the absence of TD in high-altitude native Tibetan chickens. Importantly, hypoxia experiments also showed that during hypoxia, tibial angiogenesis was enhanced, which was due to pro-angiogenic factor up-regulation (including VEGFA, VEGFR1, VEGFR2, and IL-8), in AACs. Moreover, we observed that thiram-induced TD could strongly inhibit tibial angiogenesis in the hypertrophic zone through coordinated down-regulation of HIF-1α and pro-angiogenic factors, leading to a disruption in the blood supply to the TGP. Taken together, these findings reveal that the occurrence of TD is highly associated with inhibition of tibial angiogenesis through down-regulated expression of HIF-1α, VEGFA and VEGF receptors, which results in suppression of TGP development.

## Introduction

Normal development of the bone is very important in meat-type broilers. Abnormal development of the bone (such as tibial dyschondroplasia, rickets, femoral head necrosis, and perosis) can lead to severe economic losses to the poultry industry and directly compromise poultry welfare^1–5^. Although these problems are of great concern for researchers around the world, the underlying etiology of tibial dyschondroplasia (TD) is not fully understood^5–8^.

TD is an abnormal development of the tibia that is characterized by the accumulation of non-mineralized and avascular cartilage, which is attributable to tibial chondrocyte death due to a low or absent blood supply that results in deformed bones and lameness^4, 7, 9^. Meanwhile, these consequences highly increase the rates of mortality and morbidity. Broilers suffering from TD are likely susceptible to fractures during the feeding process, which can contribute to an enormous economic loss for farmers and influence animal welfare^10^. Numerous studies have reported that TD is highly related to abnormal ossification and elongation of the tibial growth plates due to suppressed chondrocyte proliferation and differentiation. However, the mechanisms underlying the etiology of TD associated with the development of the growth plate are not well understood^11, 12^.

During the development of the tibia, endochondral ossification is initiated to achieve the bone length, and the bone is then converted to the bony tissue that occurs at the growth plate cartilage and adjacent metaphysis^2, 13^. During this process, chondrocytes in the resting zone (RZ) of the tibial growth plate are activated to proliferate in the proliferative chondrocyte zone (PZ). The chondrocytes subsequently progress to maturation and eventually undergo apoptosis in the hypertrophic chondrocyte zone (HZ), which is the main contributor to bone growth; this process is followed by invasion of blood vessels from the perichondrium^14, 15^. Importantly, the invading vasculature not only triggers the calcified hypertrophic cartilage matrix and formation of the bone marrow cavity but also recruits osteoblast and osteoclast precursors that are converted to bone trabecula; eventually, cartilage is replaced by mineralized bone deposits^13–17^. This program of tibial development is regulated by a complex molecular mechanism, including the mechanistic target of rapamycin (mTOR) pathway, PI3K/AKT/SMAD1 pathway, hypoxia-inducible factor (HIF)-1α pathway and vascular endothelial growth factor (VEGF)-VEGF receptor (VEGFR) pathway^15, 18–20^.

Our previous study showed that hypoxia has a potential new role in increasing the vascular formation of proximal tibial growth plates to strengthen and enhance the sizes of the growth plates, which eventually promotes the development of bone^5^. Moreover, Genin *et al*. also indicated that the absence of a vasculature in the lesions of the TD growth plates may be involved in the etiology of the disease, and the expression of HIF-1α in particular is essential for chondrogenesis^10^. HIF-1α is the major regulator of the hypoxia responses, which can activate the downstream genes of VEGFA and even the expression of VEGF receptors (VEGFR1/2), while VEGFA is a master regulator of angiogenesis and appears to be the most selective angiogenic factor acting on endothelial cells^21–24^. In addition, interleukin-8 (IL-8), a pro-angiogenic factor, is also a potent inducer of angiogenesis^24, 25^. Although vascular formation in the tibial growth plates has been shown to be essential for tibial chondrocyte proliferation and bone development, the underlying molecular mechanisms of angiogenesis in tibial development remain unclear^4, 9^. Furthermore, we observed that the activation of VEGFA and its receptors in chondrocytes via up-regulation of HIF-1α could stimulate the growth and development of blood vessels to promote normal development of the tibial growth plates under hypoxic conditions. This may be associated with the absence of TD in Tibetan chickens (an indigenous poultry breed living in a high-altitude hypoxic environment)^5, 26^. Therefore, in this study, we employed a thiram-induced TD model, which is commonly used to study the poultry TD model, to investigate the impact of inhibition of angiogenesis-related genes in regulating angiogenesis and its underlying mechanisms.

## Results

### The Tibetan chicken and its tibia grow slowly compared to the AA chicken

**Figure 1:**
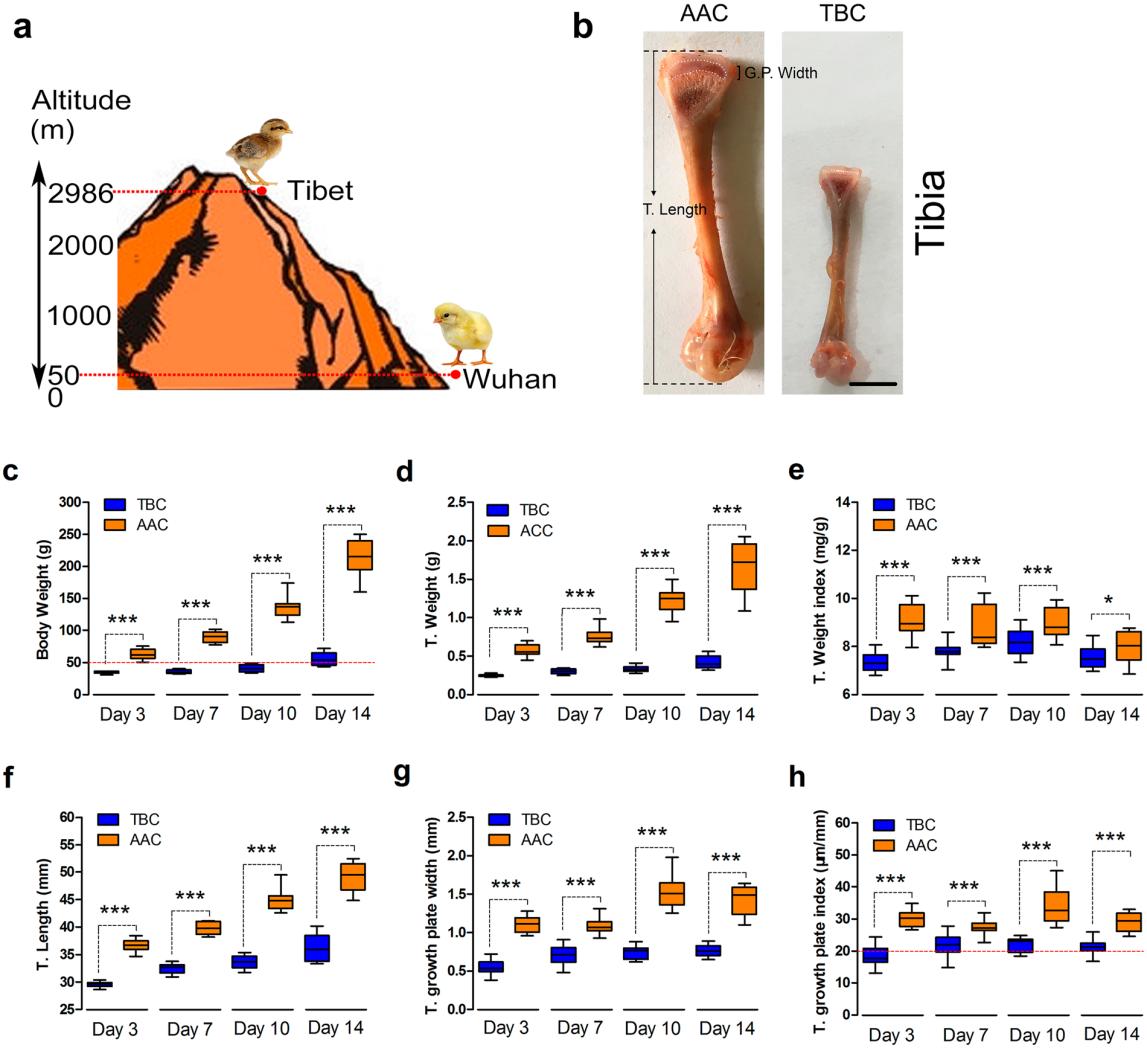
Tibetan chickens and their tibiae grow slowly compared to AA chickens. (**a**) Feeding location (experimental site) of the TBCs (2,986 meters above sea level; Tibet, China) and AA chickens (50 meters above sea level; Wuhan, China). (**b**) Standard for measurement of the T. Length (tibia length) and T. G. P. Width (tibial growth plate width). (**c**,**d**,**f**,**g**) The body weights and tibia weights of the chickens and the lengths and growth plate widths of the tibiae were recorded and compared between the TBCs and AACs. (**e**) The tibial weight index was determined as the tibial weight per corresponding body weight of the chicken (T. Weight/body weight, mg/g) between the TBCs and AACs. (**h**) The tibial growth plate index was determined as the tibial growth plate width per tibia length (T. growth plate width/T. length, µm/mm) between the TBCs and AACs. Student’s *t*-test, ^***^ *p* < 0.001, n = 16; Error bars indicate SD. AAC, Arbor Acres chicken; TBC, Tibetan chicken.

As shown in Fig. 1a, the Tibetan chicken (TBC), living at 2,986 meters above sea level, is an indigenous breed of poultry with adaptability to high-altitude hypoxic environments^5, 26^. We found that the BWs (body weights), T. Weights and T. Weight indices of TBCs were significantly lower than those of lowland chickens (Arbor Acres chickens, AACs; 50 meters above sea level) at the ages of 3, 7, 10, and 14 days (Fig. 1a,c–e). Additionally, our study also found that the length, growth plate, and growth plate index (T. growth plate width/T. length, µm/mm) of the tibiae were clearly lower in TBCs than in AACs during the experimental period (*p* < 0.001, *p* < 0.001, and *p* < 0.001 respectively; Fig. 1b,f–h). These results demonstrated that the TBC is a unique breed with slow growth and a small size, which may be related to the local hypoxic climate.

### Higher tibial vascular distribution in the hypertrophic chondrocyte zones of Tibetan chickens

**Figure 2:**
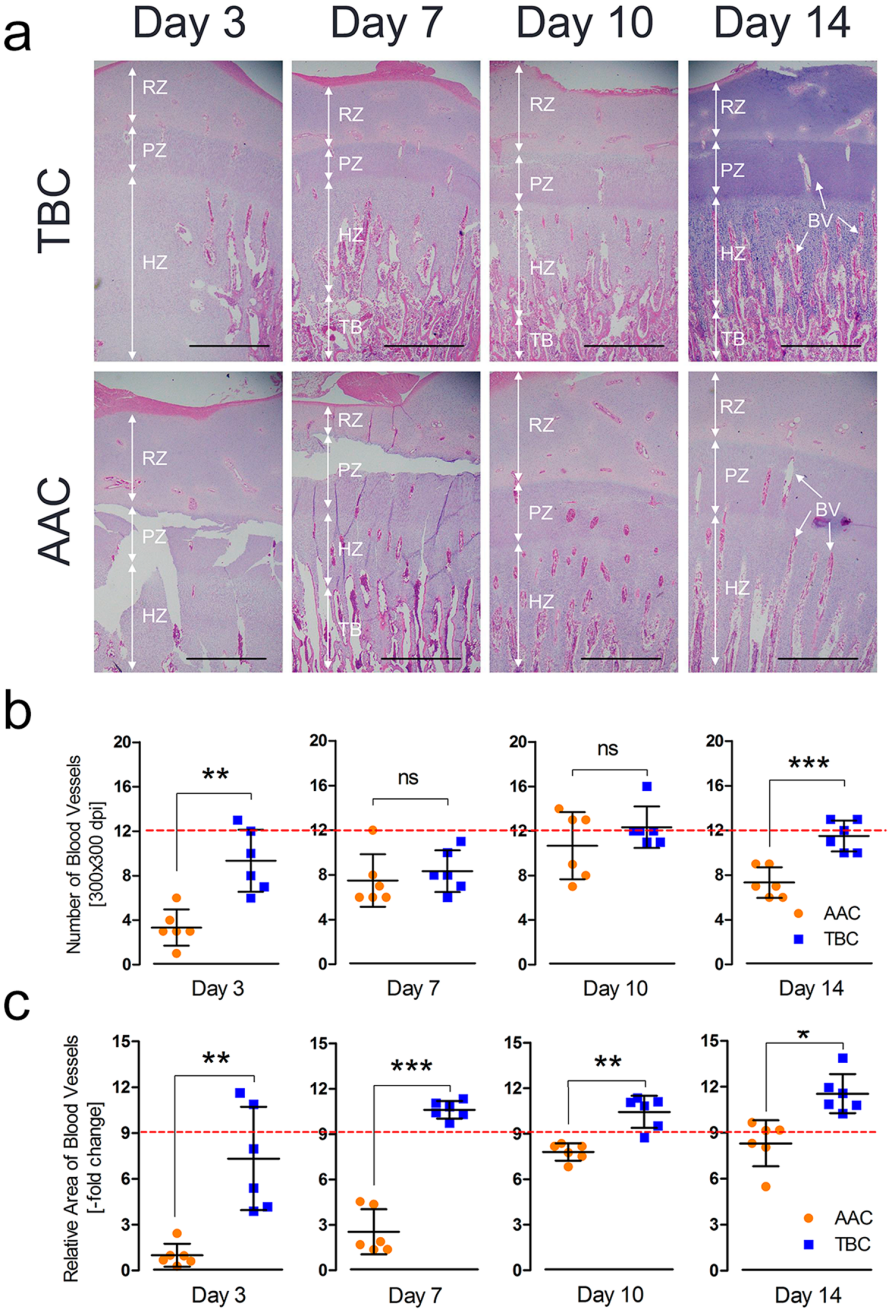
Higher tibial vascular distribution in the hypertrophic chondrocyte zones of TBCs. (**a**) Tibial vascular distribution was significantly increased in TBCs compared to AACs. The arrows indicate BVs (blood vessels); RZ, resting chondrocyte zone; PZ, proliferative chondrocyte zone; HZ, hypertrophic chondrocyte zone; and TB, trabecular bone. Scale bar = 500 µm. (**b**,**c**) The relative BV area and BV number were determined from two isolated groups using Image-Pro® Plus 6.0. Student’s *t*-test, ^*^ *p* < 0.05, ^**^ *p* < 0.01, ^***^ *p* < 0.001, n = 6; Error bars indicate SD. ns, not significant. AAC, Arbor Acres chicken; TBC, Tibetan chicken.

In addition to being a slow-growing poultry breed, the TBC has no reported leg disorders, particularly TD^5^. The mechanism underlying the unique tibial structure of TBCs is still unclear. To explore the difference in tibial structure between high-altitude TBCs and lowland AACs, histological examinations were performed to compare the vascular distributions in the hypertrophic zone of the tibia growth plates of TBCs and AACs. In this study, we observed that the vascular distributions of TBCs increased over two weeks compared with AACs, as shown in Fig. 2a. Simultaneously, we found that the relative blood vessel area was increased on day 3, day 7, day 10, and day 14 (*p* = 0.005, *p* < 0.001, *p* = 0.003, and *p* = 0.032, respectively) and that the blood vessel number was also elevated on day 3 and day 14 (*p* = 0.002 and *p* < 0.001, respectively) in the hypertrophic chondrocyte zones of the tibiae of TBCs (histological pictures as shown in Fig. 2b,c). These results suggest that the higher tibial vascular distribution in the hypertrophic chondrocyte zones of TBCs may be the basis of the absence of TD in this indigenous breed.

### Higher expression of angiogenesis-related mRNAs and proteins in Tibetan chickens

**Figure 3:**
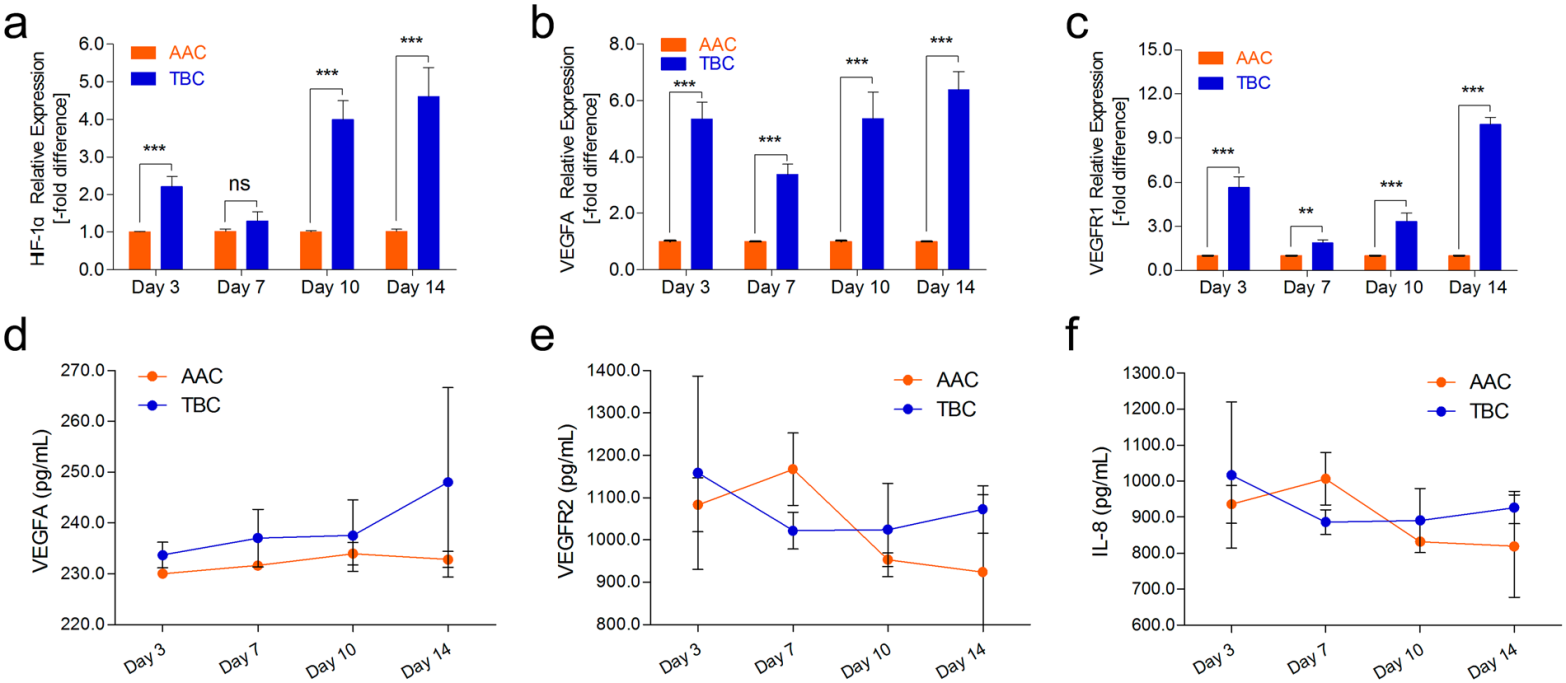
Higher levels of angiogenesis-related mRNA and protein in TBCs. (**a**–**c**) The mRNA expression levels of HIF-1a, VEGFA, and VEGFR1 were determined by qRT-PCR. The mRNA expression levels of the AAC were normalized to 1. GAPDH was used as the loading control. The results are representative of at least three independent experiments, which were run in triplicate, and are expressed as the mean ± SD. Student’s *t*-test, ^*^ *p* < 0.05, ^***^ *p* < 0.001, n = 3. (**d**–**f**) The serum protein concentrations of VEGFA, VEGFR2, and IL-8 were measured using an ELISA kit. Student’s *t*-test, n ≥ 3; Error bars indicate SEM. AAC, Arbor Acres chicken; TBC, Tibetan chicken.

Next, we determined whether angiogenesis-related genes and proteins were also altered in TBCs during the experimental period. The expression levels of HIF-1a, VEGFA, VEGFR1, VEGFR2, and IL-8 in the TBCs and AACs were measured by qRT-PCR and ELISA using tibial tissues and sera, respectively. As shown in Fig. 3a–c, significantly up-regulated expression levels of HIF-1a (except on day 7), VEGFA, and VEGFR1 were observed in the tibial growth plates of TBCs compared to AACs. Additionally, the protein concentrations of VEGFA, VRGFR2, and IL-8 in the TBCs were clearly higher than in the AACs by ELISA, especially on day 14 of the experiment (Fig. 3d–f). These results collectively suggest that TBCs have up-regulated angiogenesis-related mRNA and protein expression levels, along with increased HIF-1a, which promote vascular distribution or vessel formation in the tibia in response to possible adaptation to the high-altitude hypoxic environment.

### Hypoxia increases tibial vascular formation in the hypertrophic chondrocyte zone

**Figure 4:**
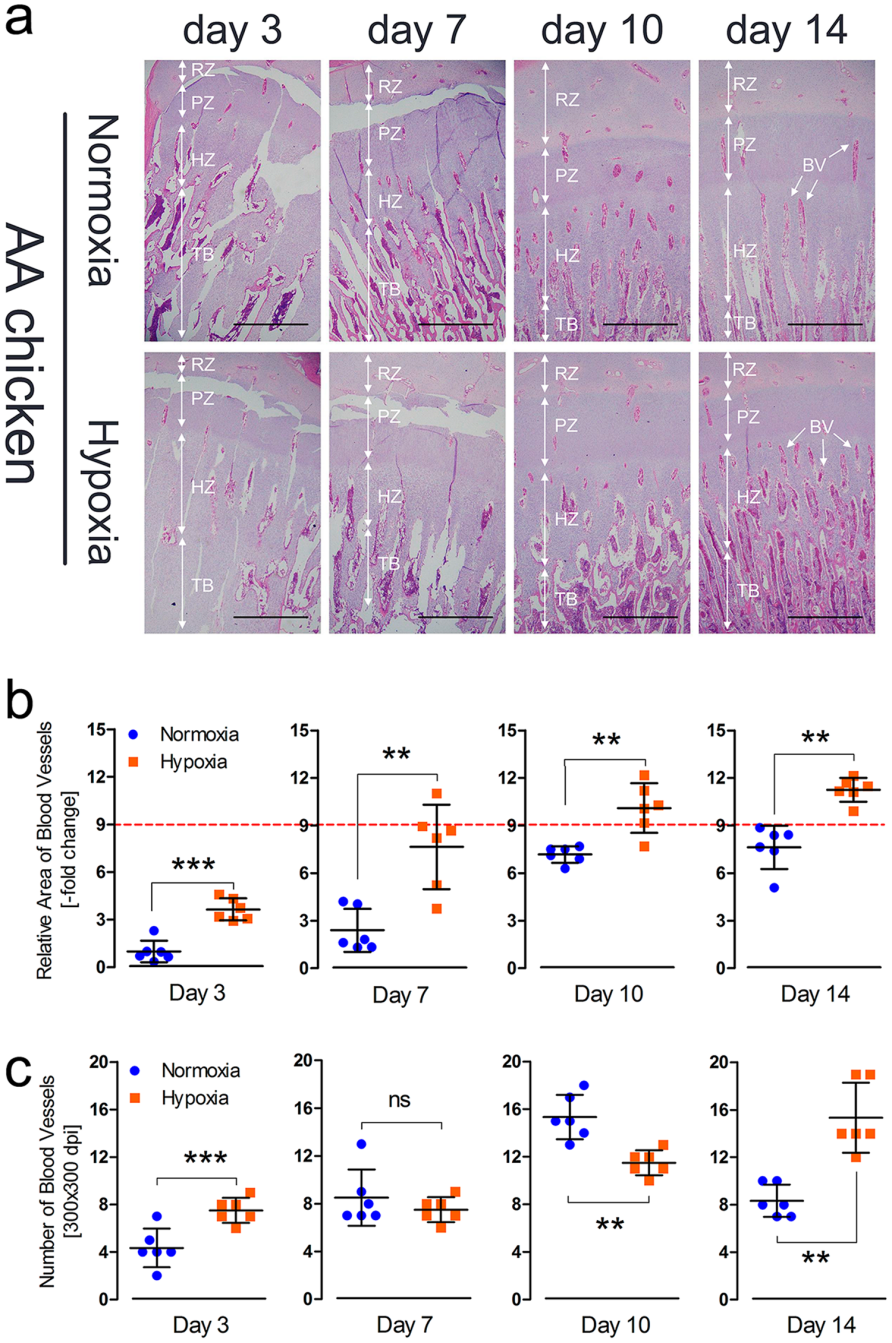
Hypoxia increases tibial vascular distribution in the hypertrophic chondrocyte zone. (**a**) Tibial vascular distribution clearly increased in the hypoxia group compared to the normoxia group. The arrows indicate the BVs (blood vessels); RZ, resting chondrocyte zone; PZ, proliferative chondrocyte zone; HZ, hypertrophic chondrocyte zone; and TB, trabecular bone. Scale bar = 500 µm. (**b**,**c**) The relative BV area and number were determined from two isolated groups using Image-Pro® Plus 6.0. The value for the red line as the reference in this figure is 9. Student’s *t*-test, ^**^ *p* < 0.01, ^***^ *p* < 0.001, n = 6; Error bars indicate SD. ns, not significant.

To further determine the effect of hypoxia on vascular development in the tibia, AACs reared at high-altitude were evaluated to compare the tibial vascular distributions of the normoxia- and hypoxia-treated groups. We observed that the vascular distribution in the hypoxic group was clearly enhanced, especially on day 14, compared to normoxia group, as shown in Fig. 4a. Simultaneously, we also found that the relative area of the blood vessels was significantly increased on day 3, day 7, day 10, and day 14 (*p* < 0.001, *p* = 0.002, *p* = 0.010, and *p* = 0.002, respectively) and that the number of blood vessels was also markedly elevated on day 3, day 10 and day 14 (*p* < 0.001, *p* = 0.008 and *p* = 0.007, respectively) in the hypertrophic chondrocyte zones of the tibiae in the hypoxia group, by statistical analysis of histological pictures, as shown in Fig. 4b,c. Thus, these observations demonstrated that the tibial vascular distribution was enhanced under hypoxic conditions.

### Hypoxia promotes angiogenesis-related mRNA and protein expression

**Figure 5:**
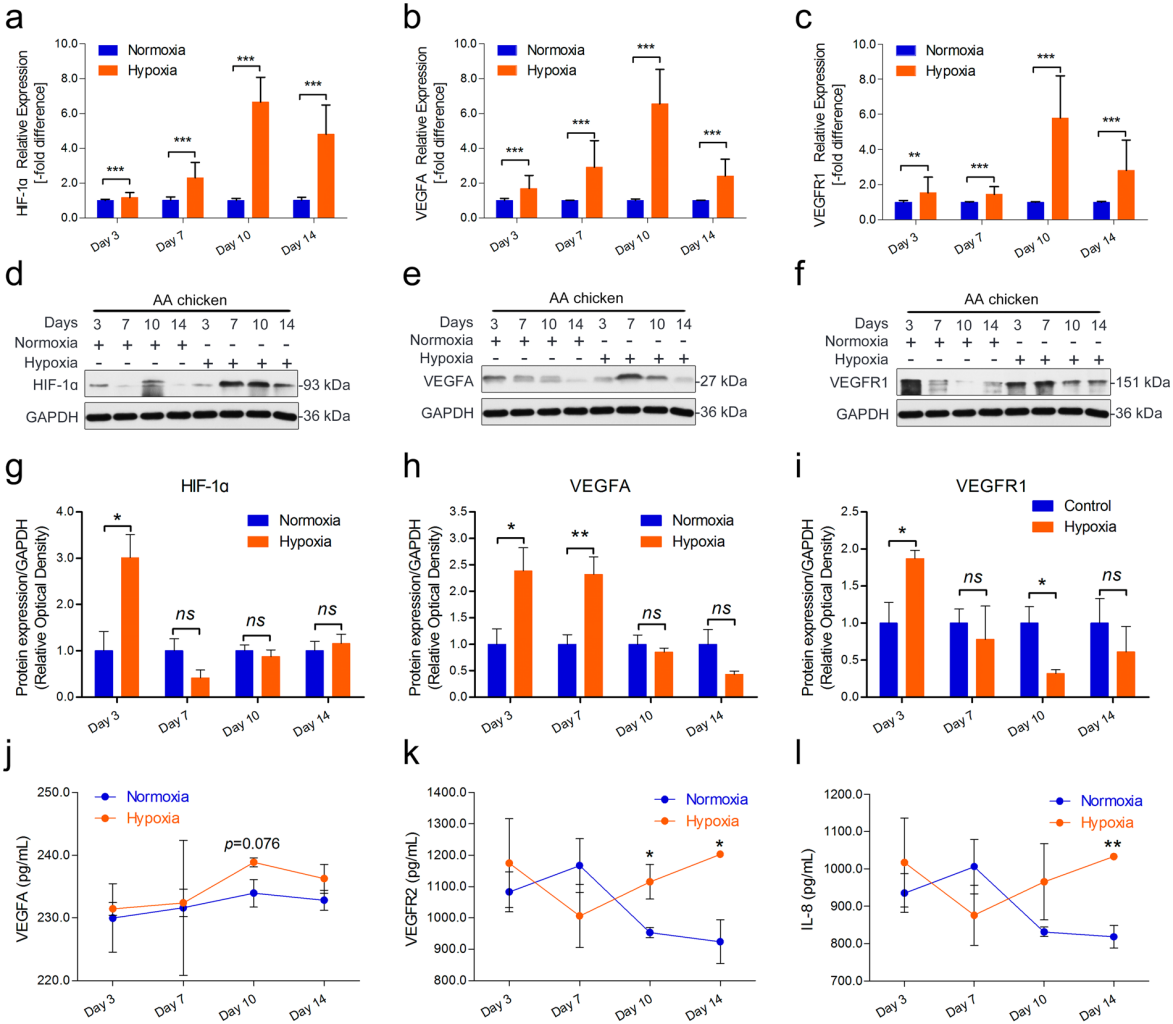
Hypoxia enhances angiogenesis-related mRNA and protein expression. (**a**–**c**) The mRNA expression levels of HIF-1α, VEGFA, and VEGFR1 were determined by qRT-PCR. mRNA expression levels from the normoxia group were normalized to 1. GAPDH was used as the loading control. The results are representative of at least three independent experiments, which were run in triplicate, and are expressed as the mean ± SD. Student’s *t*-test, ^*^ *p* < 0.05, ^***^ *p* < 0.001, n = 3. (**d**–**f**) The protein expression levels of HIF-1α, VEGFA, and VEGFR1 were determined by Western blotting. (**g**–**i**) The results of the western blots were quantified and analyzed using Image-Pro® Plus 6.0. Student’s *t*-test, ^*^ *p* < 0.05, ^**^ *p* < 0.01, n = 3; Error bars indicate SEM. ns, not significant. (**j**–**k**) The serum concentrations of VEGFA, VEGFR2, and IL-8 were measured using an ELISA kit. Student’s *t*-test, n ≥ 3; Error bars indicate SEM.

A hypoxic environment has been known to be essential for enhanced vascular distribution in tibial growth plates. To explore the link between hypoxia-induced downstream gene activity and angiogenesis in the tibia under hypoxic conditions, qRT-PCR was performed to compare the gene and protein expression levels between the hypoxia group and normoxia group in AACs. As shown in Fig. 5a–c, we noticed up-regulation of the HIF-1a, VEGFA, and VEGFR1 mRNA expression levels under hypoxic conditions in the tibial growth plates. Subsequently, Western blot and statistical analyses of these results were performed to compare the protein expression levels between the hypoxic group and normoxia group in AACs. As shown in Fig. 5d–f, significant increases in the secretion of the HIF-1a protein, VEGFA protein, and VEGFR1 protein were observed in the tibia of the hypoxia group compared to the tibia of the normoxia group. In addition, statistical analysis of Western blotting revealed a statistically significant up-regulated expression level of the HIF-1a protein on day 3 (*p* = 0.033), the VEGFA protein on day 3 and day 7 (*p* = 0.036 and *p* = 0.005, respectively), and the VEGFR1 protein on day 3 and day 10 (*p* = 0.029 and *p* = 0.039, respectively) in the tibial growth plates of the hypoxia group compared to the normoxia group (Fig. 5g–i). To further confirm the protein levels of VEGFA, VEGFR2, and IL-8 under hypoxic conditions, ELISA was performed to compare the serum protein levels in the hypoxia and normoxia AAC groups. As shown in Fig. 5j–l, the serum protein concentration of VEGFA was slightly enhanced on day 10 (*p* = 0.076), that of VEGFR2 was significantly increased on day 10 and day 14 (*p* = 0.049 and *p* = 0.016, respectively), and that of IL-8 was markedly enhanced on day 14 (*p* = 0.002) under hypoxia compared to normoxia. Collectively, these results suggest that up-regulation of angiogenesis-related mRNA and protein expression along with HIF-1a is higher during hypoxia than during normoxia. These results suggested that hypoxic conditions mediated the expression of HIF-1a to activate downstream genes, namely, VEGFA and VEGF receptors, which promote vessel formation in the tibia.

### Thiram suppresses tibial growth and increases the T. growth plate width

**Figure 6:**
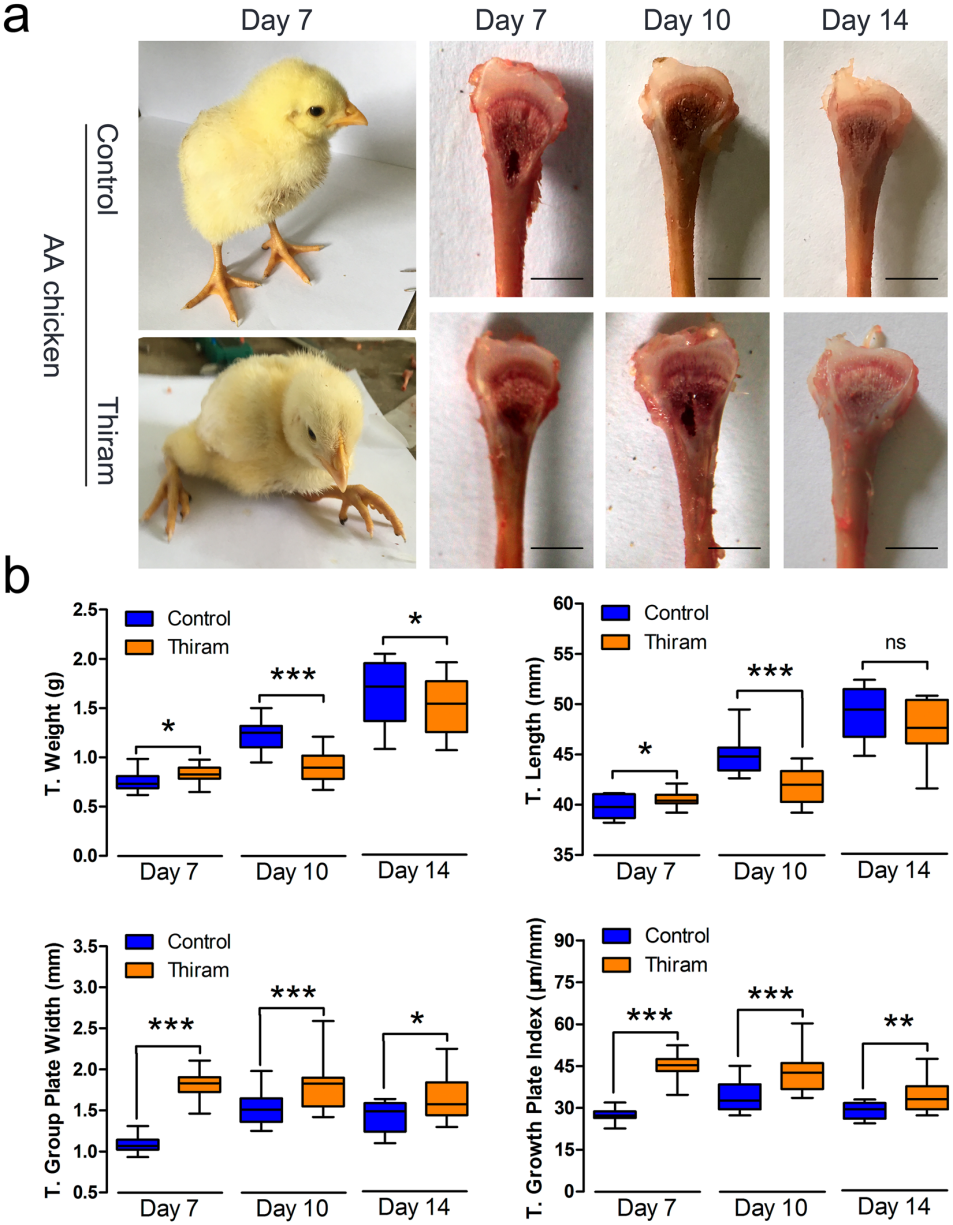
Thiram diminishes the tibial growth and increases T. growth plate width. (**a**) Thiram-treated AA chickens reveal lameness in the broiler and an increased width of the growth plate. (**b**) Weights, lengths, and growth plate widths of the tibiae were recorded, and the tibial growth plate index was determined as the tibial growth plate width per tibia length (T. growth plate width/T. length, µm/mm) between the thiram-induced group and the control group. Student’s-*t* test, ^*^ *p* < 0.05, ^**^ *p* < 0.01, ^***^ *p* < 0.001, n = 16; Error bars indicate SD. ns, not significant.

The above findings show that the expression of both HIF-1a and angiogenesis-related genes was increased after the induction of hypoxia, which may be associated with the absence of TD in TBCs. However, the mechanism responsible for the angiogenesis-related gene dynamics in thiram-induced TD is unknown. To this end, a thiram-induced TD animal model, which exhibited distinct lameness, was used (Fig. 6a). First, the tibial morphological results showed that the width of the proximal tibial growth plates of the AACs were markedly enlarged in the thiram-induced group (Fig. 6a). Additionally, our study found that the T. weights of the AACs in the thiram-treated group were significantly lower than those of the control group at the ages of 7, 10, and 14 days (*p* < 0.001; Fig. 6b). At the same time, statistical measurements indicated that the T. lengths on day 7 and day 10 (*p* = 0.050 and *p* < 0.001, respectively) and the T. growth plate widths on day 7, day 10, and day 14 (*p* < 0.001, *p* < 0.001, and *p* = 0.031, respectively) were visibly lower and that the T. growth plate indices (T. growth plate width/T. length, µm/mm) on day 7, day 10, and day 14 (*p* < 0.001, *p* < 0.001, and *p* = 0.005, respectively) were significantly higher in the thiram-treated group than in the control group, as shown in Fig. 6b. These results demonstrate that thiram can induce the occurrence of TD and inhibit tibial growth in AACs.

### Thiram suppresses tibial vascular distribution in the hypertrophic chondrocyte zone of AA chickens

**Figure 7:**
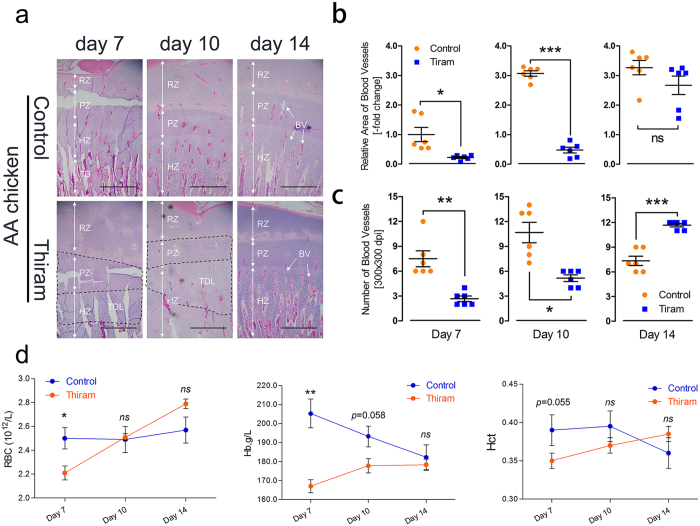
Thiram suppresses tibial vascular distribution in the hypertrophic chondrocyte zones of AACs. (**a**) Tibial vascular distribution was significantly inhibited in the Thiram-treated group compared to the control group. The arrows indicate the BVs (blood vessels); TDL, tibial dyschondroplasia lesion; RZ, resting chondrocyte zone; PZ, proliferative chondrocyte zone; HZ, hypertrophic chondrocyte zone; and TB, trabecular bone. Scale bar = 500 µm. (**b**,**c**) The relative BV area and number were determined from two isolated groups using Image-Pro® Plus 6.0. Student’s *t*-test, ^*^ *p* < 0.05, ^**^ *p* < 0.01, ^***^ *p* < 0.001, n = 6; Error bars indicate SD. ns, not significant. (**d**) RBC (red blood cell) counts, Hb (hemoglobin) levels and Hct (hematocrit) values were determined for all blood samples. Student’s *t*-test, ^*^ *p* < 0.05, ^**^ *p* < 0.01, n = 6; Error bars indicate SEM. ns, not significant.

To further determine the effect of thiram on vascular development in the tibia, AAC was treated with Thiram to compare the vascular distribution of the tibia in the control group to that of the thiram-treated group. We observed that the vascular distribution in the thiram-treated group was dramatically decreased, especially on day 7 and day 10, and presented a clear TDL (tibial dyschondroplasia lesion) zone compared to the control group (Fig. 7a). Simultaneously, we statistically determined that the relative area of the blood vessels was significantly diminished on day 7 and day 10 (*p* = 0.022 and *p* < 0.001, respectively) and that the number of blood vessels on day 7 and day 10 (*p* = 0.004 and *p* = 0.014, respectively) was also markedly decreased. However, on day 14, the number of blood vessels was dramatically elevated (*p* < 0.001) in the hypertrophic chondrocyte zones of the tibiae in the thiram-treated group, as shown in Fig. 7b,c. Similarly, the complete blood counts (CBCs) showed that the total RBC counts, Hb concentration and Hct values on day 7 in the thiram-treated group were clearly lower (*p* = 0.031, *p* = 0.001, and *p* = 0.055, respectively) and that these values had a subsequent rising trend in comparison to the control group (Fig. 7d). These observations revealed that the tibial vascular distribution and CBC indices were severely suppressed in the thiram-treated group.

### Thiram inhibits angiogenesis-related mRNA and protein expression in the tibia

**Figure 8:**
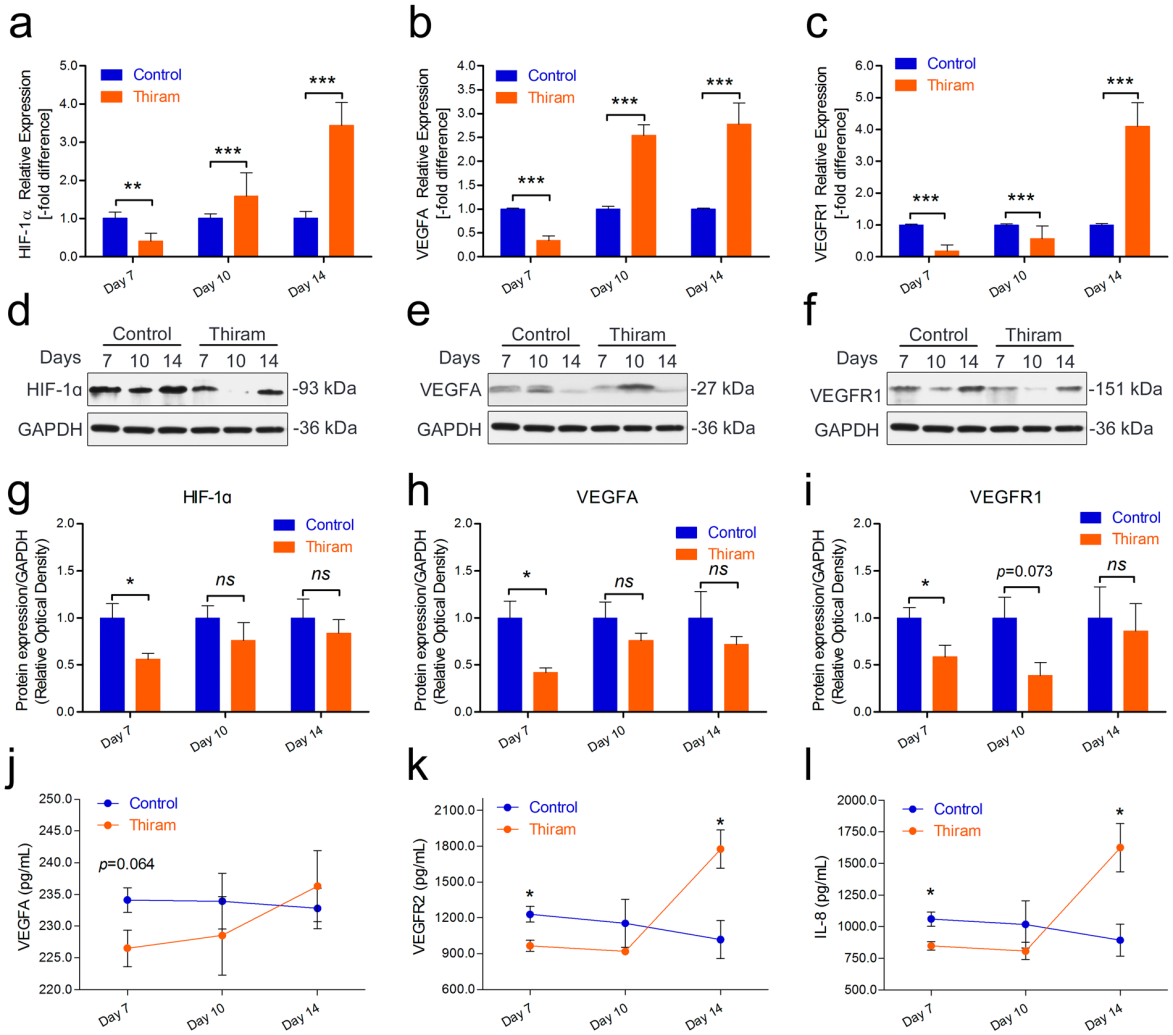
Thiram inhibits angiogenesis-related mRNA and protein expression. (**a**–**c**) The mRNA expression levels of HIF-1a, VEGFA, and VEGFR1 were determined by qRT-PCR. mRNA expression levels from the control group were normalized to 1. GAPDH was used as the loading control. The results are representative of at least three independent experiments, which were run in triplicate, and are expressed as the mean ± SD. Student’s *t*-test, ^**^ *p* < 0.01, ^***^ *p* < 0.001, n = 3. (**d**–**f**) The protein expression levels of HIF-1a, VEGFA, and VEGFR1 were determined by Western blotting. (**g**–**i**) The results of the western blots were quantified and analyzed using Image-Pro® Plus 6.0. Student’s *t*-test, ^*^ *p* < 0.05, ^**^ *p* < 0.01, n = 3; Error bars indicate SEM. ns, not significant. (**j**,**k**) The serum concentrations of VEGFA, VEGFR2, and IL-8 were measured using ELISA kits. Student’s *t*-test, n ≥ 3; Error bars indicate SEM.

**Figure 9:**
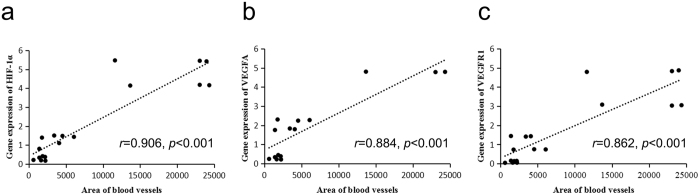
Evaluation of the correlation between the blood vessel area and gene expression. A positive correlation in HIF-1α (**a**), VEGFA (**b**) and VEGFR1(**c**) were found with Pearson’s test between the average blood vessel area (n = 6) and average gene expression level in the tibia (n = 3) during the experiment.

Lastly, to explore the link between thiram-induced gene activity and angiogenesis in the tibia, qRT-PCR was performed to compare gene expression levels between the thiram-treated and control AAC groups. As shown in Fig. 8a–c, we observed marked down-regulation of the HIF-1a, VEGFA, and VEGFR1 mRNA expression levels on day 7 (*p* < 0.001); these levels were significantly elevated on day 10 and day 14 (except VEGFR1 on day 10) in the thiram-treated group in the chicken tibial growth plates. Moreover, Western blotting and statistical analyses of these results were performed to compare the protein expression levels of the thiram-treated and control AAC groups. As shown in Fig. 8d–f, significantly down-regulated expression of the HIF-1a protein, VEGFA protein, and VEGFR1 protein were observed in the tibiae of the thiram-treated group compared to the tibiae of the control group. Meanwhile, a statistical analysis of the Western blotting results revealed that the expression levels of the HIF-1a protein on day 7 (*p* = 0.047), VEGFA protein on day 7 (*p* = 0.039), and VEGFR1 protein on day 7 (*p* = 0.041) were significantly diminished in the tibial growth plates of the thiram-treated group compared to those of the control group (Fig. 8g–i). Importantly, to further confirm the changes in the serum protein levels of VEGFA, VEGFR2, and IL-8, ELISA was performed to compare the serum protein concentrations of the thiram-treated and control AAC groups. In the thiram-treated group, the serum protein concentration of VEGFA was clearly lower on day 7 (*p* = 0.076), that of VEGFR2 was significantly lower on day 7 (*p* = 0.049 and *p* = 0.016, respectively) and that of IL-8 was markedly lower on day 7 (*p* = 0.002) compared to the control group, as shown in Fig. 8j–l. In addition, the expression levels of HIF-1a, VEGFA and VEGFR1 in the tibial growth plates were strongly correlated with the blood vessel area in the corresponding tibial hypertrophic zones (*r* = 0.906, *r* = 0.884, and *r* = 0.8622, respectively) (Fig. 9a–c). These results suggest that thiram inhibits the expression of HIF-1a and angiogenesis-related mRNAs and proteins and that this inhibition is the most direct cause of TD in chickens, due to inhibition of tibial angiogenesis, which blocks the supply of nutrients caused by chondrocyte death.

## Discussion

Tibial dyschondroplasia (TD) is a disorder that affects broiler and turkey growth and results in abnormal form of the tibia with the appearance of non-vascularized and non-mineralized cartilage in the tibial growth plates^5, 27–29^. TD is one of the most challenging conditions to treat because of the unknown etiology of this disease. We noticed that evidence of TD in Tibetan chickens, an aboriginal breed with a several-thousand-year history of living on the Qinghai - Tibet Plateau where the oxygen content is low, has never been presented, which may be due to the good relationship between these chickens and the high-altitude environment^30^.

The comparative results of the BW and tibia parameters showed that the BWs, T. Weights, T. lengths, and T. growth plate widths of the TBCs were markedly lower than those of the AACs. Additionally, the T. Weight index and T. growth plate width index were also lower, suggesting that the slow growth of TBCs may be related to the local hypoxic climate^31^ (Fig. 1c–h). These observations were consistent with a previous report by Gao *et al*. regarding inhibition of body weight gain under hypoxic conditions^32^. We know that TD is strikingly characterized by an avascular growth plate^8^. Impaired functioning of the bone blood vessels may be associated with the occurrence of some skeletal diseases (i.e., osteonecrosis, osteoporosis)^33^. However, the current study observed that TBCs have higher tibia vascular distributions in their hypertrophic chondrocyte zones compared to AACs, which may be directly linked to the absence of TD in TBCs (Fig. 2). Meanwhile, we found that genes and proteins that are involved in vessel formation (including HIF-1α, VEGFA, VEGFR1, VEGFR2, and IL-8) were up-regulated in the tibial growth plates of the TBCs (Fig. 3). These data provide evidence showing that, compared to AACs, TBCs have higher vascular distributions (vessel formation) in their tibial growth plates, which contributes to oxygen and nutrition delivery to the tibia due to long-term adaptation to the hypoxic environment. These observations provide new insights into TD^34^.

To further determine the effect of hypoxia on vascular development, which promotes oxygen and nutrition delivery to tissues, in the tibial growth plates, we proposed hypoxia-induced experiments in AACs at high altitude^34^. Numerous studies have reported that VEGFA and its receptors (including VEGFR1 and VEGFR2) are pro-angiogenic cytokines that sustain angiogenesis in tumors^18, 21, 23, 24, 35–37^. However, there are few reports regarding bone development in chickens^9^. Here, we have presented strong evidence indicating that tibial angiogenesis in the hypertrophic chondrocyte zone is enhanced by hypoxia in AACs (Fig. 4) and that up-regulation of angiogenesis-related mRNA and protein expression (VEGFA and its receptors) is evident with hypoxia (Fig. 5a–i). Moreover, HIF-1α and the concentrations of VEGFA, VEGFR2, and IL-8 were also markedly enhanced (Fig. 5j–l) under hypoxia. An investigation of thyroid carcinoma angiogenesis by Daniell *et al*. indicated that regulation of the HIF-1 and VEGF-dependent pathway modulates angiogenesis^38^. Furthermore, Yin *et al*. also showed that up-regulation of VEGF by HIF-1α plays an important role in triggering angiogenesis to protect myocardial cell survival in a hypoxic microenvironment^39^. The results of this study further clarify that hypoxia can mediate the expression of HIF-1a to activate downstream genes, namely VEGFA and VEGF receptors, in the tibial growth plate to promote vessel formation. These data enable a better understanding of the potential new role of hypoxia in tibial growth plate development^5^. Once again, these observations may be explained by hypoxia-induced angiogenesis in the tibial hypertrophic chondrocyte zone, as hypoxia-induced angiogenesis is highly associated with the absence of TD in TBCs.

Although the VEGF/VEGFR signaling pathway has been shown to be essential for vessel formation in the tibial growth plate under hypoxia, the underlying mechanisms of the specific role of this pathway in tibial chondrocyte proliferation and development are unclear^9, 38, 39^. Next, thiram-induced TD in AACs was evaluated, and we found that thiram could inhibit the development of the tibia and increase tibial growth plate width susceptibility to fractures, as shown in Fig. 6. In addition, similar to our previous reports, we showed that the tibial vascular distribution was substantially decreased in the hypertrophic chondrocyte zone after the thiram treatment, whereas new vessel formation was observed when thiram was removed from the broiler diet^8, 40^ (Fig. 7a–c). Interestingly, the complete blood counts showed similar results where the total RBC counts, Hb levels and Hct levels were markedly decreased on day 7 (Fig. 7d). Many researchers have shown that the bone marrow is the major site of erythropoiesis in humans and mice^41–43^. Additionally, a low Hb content per RBC is the result of an insufficient iron content in the bone marrow during erythropoiesis^43^. This information implied that thiram suppresses bone marrow activity resulting in decreased production of RBCs and that thiram also inhibits angiogenesis by disrupting the blood supply in tibial chondrocytes, which will seriously affect growth and development of the tibial growth plate.

Many previous studies have shown that HIF-1α, a master regulator of the cellular response to hypoxia, is essential for growth and survival of growth plate chondrocytes *in vivo*; chondrocytes lacking functional HIF-1α undergo massive cell death in the growth plate that leads to the bone narrow and exhibit less vascularization^39, 44–46^. In addition, VEGF is also a well-characterized angiogenic factor that is activated by hypoxia and an important protein in vascular formation as previous described^39, 46^. Our observations using qRT-PCR and Western blotting in the current study results, which show that thiram inhibits the expression of HIF-1a and angiogenesis-related mRNAs and proteins which then contributes to the obstruction of vessel formation, are consistent with the aforementioned observations. Moreover, the expression levels of HIF-1a, VEGFA and VEGFR1 in the tibial growth plates have a strong correlations with the blood vessel areas in the corresponding tibial hypertrophic zones (Fig. 9). Simultaneously, the serum protein levels of VEGFA, VEGFR2, and IL-8 were also significantly lower on day 7 of the thiram treatment relative the control (Fig. 8j–l). VEGF-stimulated angiogenesis is also vital for the endochondral ossification that occurs during bone development and bone repair^47^. Thus, these findings collectively suggest that the direct cause of TD in broiler chickens is inhibition of tibial angiogenesis, which blocks the supply of nutrients, and chondrocyte death through inhibition of HIF-1α and the VEGFA/VEGFR signaling pathway.

In conclusion, our results show that the expression and secretion of HIF-1α and angiogenesis-related genes in tibial growth plates are inhibited by thiram, leading to suppression of vessel formation in the hypertrophic chondrocyte zone through coordinated down-regulation of HIF-1α and the VEGF/VEGFR signaling pathway, which are closely associated with TD. Conversely, the absence of TD in Tibetan chickens is highly associated with high-altitude hypoxia-induced angiogenesis. However, treatment of TD continues to be a major problem in the poultry industry and deserves further investigation.

## Materials and Methods

### Ethics statement

Animal experiments were approved by the Ethical Committee for Animal Research of Huazhong Agricultural University (approval permit number: 31272517) and conducted based on the state guidelines of the Standing Committee of Hubei People’s Congress, China. Before exsanguination and necropsy, injection of pentobarbital was used along with the standard protocols of euthanasia to minimize animal suffering.

### Experimental animals

**Figure 10:**
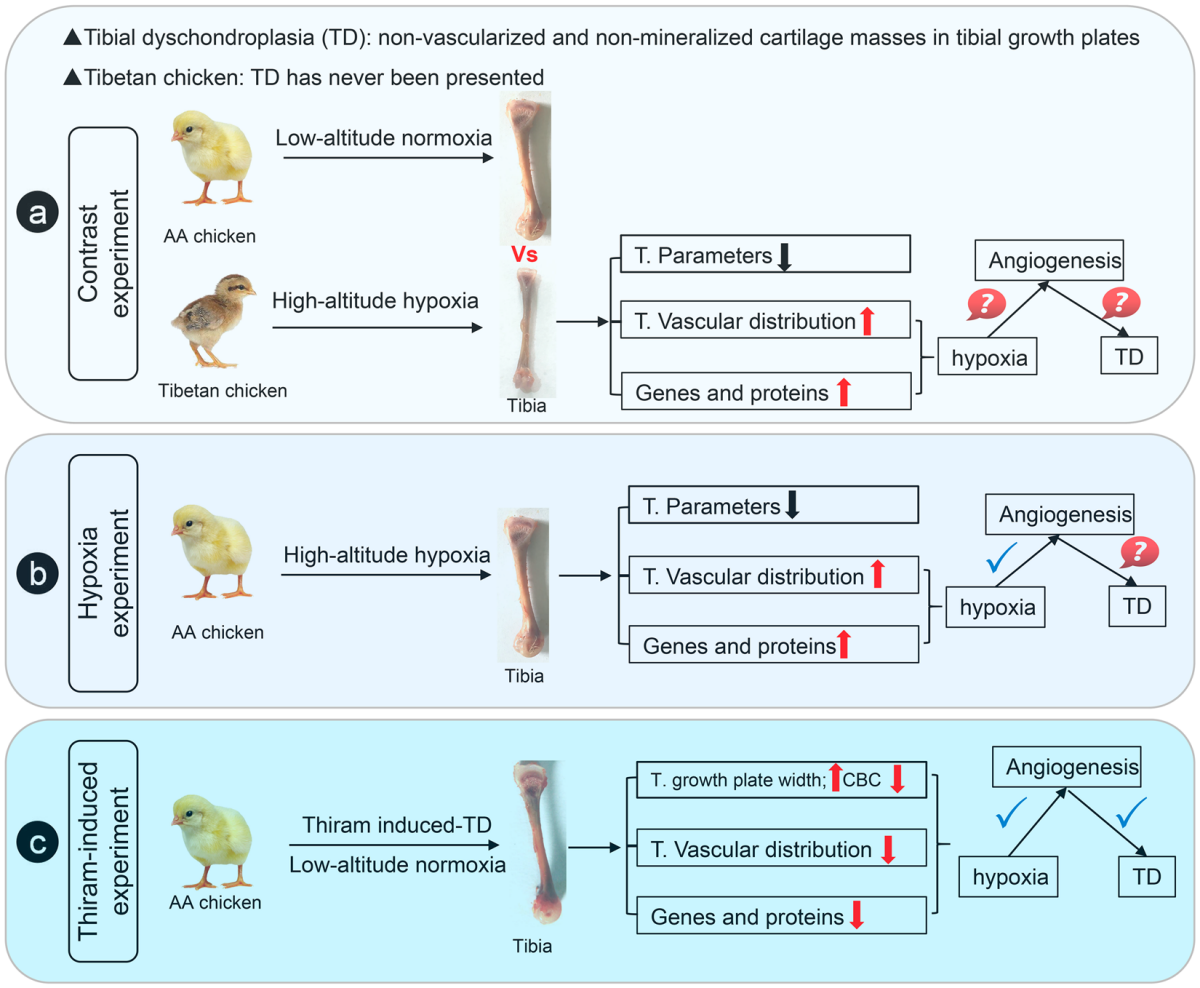
Flowchart for the experiments. (**a**) Contrast experiment of Tibetan chickens and AA chickens. (**b**) High-altitude hypoxia experiment in AA chickens. (**c**) Thiram-induced TD experiment in AA chickens.

One-day-old healthy Tibetan chickens (TBCs; n = 120) and Arbor Acres chickens (AACs; n = 120) were reared at the laboratory of the Tibet Agricultural and Animal Husbandry College (nearly 3,000 meters above sea level). In addition, one-day-old healthy Arbor Acres chickens were simultaneously reared at Huazhong Agricultural University (nearly 50 m above sea level; Fig. 10a). The chicks were reared in two-layer metal cages for 14 days. The brooding temperature was maintained from 33 °C~35 °C during the first week and steadily reduced to 29 °C at the end of second week. Daily lighting was fixed at a 23 h/1 h light/dark cycle throughout the entire experimental period. Additionally, feed and water were provided *ad libitum* ^5^.

### Hypoxia experiments

All the chicks were weighed and randomly divided equally into two groups: the hypoxia group and normoxia group (natural oxygen content and a nearly 21% oxygen content; n = 60/group, 15 chicks per cage; Fig. 10b). The oxygen content in the normoxia group was maintained using an oxygenerator (Yuwell, Suzhou, China), and oxygen levels were monitored using a gas detector (CY-7B, Oxygen Analysis Instrument Factory, Jiande, China) until the end of the experiment.

### TD establishment

The AA chickens were divided into two separate groups: the control group (normal diet) and the thiram-induced TD group (normal diet plus 50 mg/kg of thiram (tetramethyl thiuram disulphide; Macklin Biochemical Co., Ltd. Shanghai, China; Fig. 10c). The total time of the experiment was 14 days; thiram was added on day 3 and removed on day 7 following a normal diet until the end of the experiment^8^.

### Production performance statistics

The chicks were group-weighed on day 3, day 7, day 10, and day 14 with the cages. In addition, the average daily weight gain (ADG) and average daily feed intake (ADFI) were calculated and recorded per group. Feed consumption (FC) was also determined on day 3, day 7, day 10, and day 14 with the cages, and feed consumption per chick (g/chick) was calculated by dividing the total FC of each cage by the total number of chicks in the cage. The feed conversion ratio (FCR) was determined as the FC per body weight gain (g/g) per cage per unit of time. In addition, each chick that selected for the next experiment was weighed, and the value was recorded as the body weight (BW).

### Blood parameter tests

Before euthanasia, blood samples were obtained through the wing veins using heparinized syringes. The red blood cell (RBC) count, hemoglobin (Hb) level and hematocrit (Hct) values were determined for all groups. These parameters were determined using an automatic blood analyzer (XFA6000, Pulang Company, Nanjing, China).

### Tibial growth plate collection

All chickens were euthanized by cervical dislocation before injections with pentobarbital (25 mg/kg). The tibiae were retrieved, and the growth plates were detached from articular cartilages of the tibiae using a surgical knife^5, 48^. Then, the tibia was divided into two parts: one was stored in formalin, and the other was frozen in liquid nitrogen.

### Measurement of tibial parameters

On days 3, 7, 10, and 14, two chicks per treatment cage were randomly selected and sacrificed. The weight (T. weight, mg), length (T. length, mm), and growth plate width (T. growth plate width, mm) of the tibia of each chick were determined using an electronic balance (sensitive to 1 mg) and Digital Calipers (SATA91511, TATA Company, Shanghai, China). In addition, the tibial growth plate width index (T. growth plate index, determined as the T. growth plate width per T. length for each bird, µm/mm) was calculated.

### Morphology and histology of the tibial growth plates

After anesthesia by pentobarbital injection, tibial samples were obtained from 3-, 7-, 10-, and 14-day-old chicks. The stripping of the tibial longitudinal muscles and preparation of the sagittal sections of the proximal tibial growth plates were performed to analyze morphology, as previously described^5^. In addition, the tibial samples of each chick were cleared of soft tissue, fixed in 4% paraformaldehyde prior to decalcification in 10% EDTA at room temperature, and embedded in paraffin for serial 4~5-μm section preparation. In the end, histological sections were stained with hematoxylin and eosin (H&E) for microscopic examination. To analyze the blood vessel area and number in the histological results (3 chicks/group), three different microscopic fields for each picture in the hypertrophic zone adjacent to the tibial growth plate were randomly selected and counted for two isolated groups using Image-Pro® Plus 6.0.

### Quantitative Reverse Transcriptase - PCR (qRT - PCR)

Total RNA was extracted using the TRIzol reagent (Life Technologies, Carlsbad, California, USA). cDNA was synthesized using a cDNA Synthesis Kit (TransBionovo Co., Ltd, Beijing, China) per the instructions of the manufacturer. RT-qPCR was performed with the *TransStart* Tip Green qPCR Kit (TransBionovo Co., Ltd, Beijing, China) in a Step One-Plus^TM^ Real-Time PCR System (Applied Biosystems, CA, USA). All reactions were performed in triplicate. GAPDH was used as the internal control, and the relative expression of each target gene was determined using the comparative threshold cycle (C_T_) values. The sequences of the primers used were as follows: HIF-1a (forward 5′-TGAGAGAAATGCTTACAC ACAG-3′ and reverse 5′-TGATGGGTGAGGAATTGGTTCAC-3′), VEGFA (forward 5′-CGAT GAGGGCCTAGAATGTGTC-3′ and reverse 5′-AGCTCATG TGCGCTATGTGC-3′), VEGFR1 (forward 5′-TGTAACTAAGTATGCCTGTGG-3′ and reverse 5′-GGAGTTGTTGGGTATCTGC-3′), GAPDH (forward 5′-CCTCTCTGGCAAAGTCCAAG-3′ and reverse 5′-GGTCACGCTCC TGGAAGATA-3′).

### Western blotting

Tibial growth plates were homogenized in ice-cold buffer and incubated at 4 °C for 2 h. The samples were centrifuged at 12,000 rpm for 10 min to collect the supernatants (total protein), and the total protein concentrations were determined using the BCA protein quantitative detection kit (Servicebio technology, Wuhan, China). All samples were cryo-preserved at −70 °C for subsequent use. Protein samples were separated by SDS-PAGE on 12% polyacrylamide gels until the dye band reached the end of the gel and were then transferred to PVDF membranes, which were incubated in 5% skim milk at room temperature (1 h). The membranes were incubated overnight at 4 °C with the rabbit monoclonal anti-HIF-1α, anti-VEGFA and anti-VEGFR1 primary antibodies (1:1000 dilution; A11945, A5708 and A1277, respectively) (ABclonal technology, Wuhan, China). The membranes were washed 3 times with PBS-TWEEN 20 for 5 min each and were then incubated with the secondary antibody (1:3000 dilution) (HRP-labeled rabbit anti-goat secondary antibodies) for 1 h at room temperature. After washing, the bands were visualized by chemiluminescence and exposed radiography film. The images were obtained using an imaging system (EPSON, China, V300).

### Enzyme-linked immunosorbent assay (ELISA)

All blood samples were centrifuged at 3,500 rpm for 10 min, and the supernatants were stored at −80 °C until use. The serum concentrations of VEGFA (E75113), VEGFR2 (E75148), and IL-8 (E75019) for each group were measured using a chicken-specific ELISA kit according to manufacturer’s instructions (Biofine, Beijing equation Jiahong Technology Co., Ltd, China). The optical density values of each well were determined within 15 min at a wavelength of 450 nm using a Thermo Scientific Microplate Reader. Concentrations were measured in pg/mL. Each experiment was performed at least three times.

### Statistical analysis

Comparative analyses between the two groups were based on at least four independent biological experiments and were determined using Student’s *t*-test (two tailed) using the SPSS Statistics software (SPSS Version 17.0. Chicago, IL). *p* < 0.05 was considered statistically significant, and the values were presented as the means ± SD or SEM.
